# 3-Hy­droxy-2-[(2-hy­droxy-4,4-dimethyl-6-oxocyclo­hex-1-en-1-yl)(3-nitro­phen­yl)meth­yl]-5,5-dimethyl­cyclo­hex-2-en-1-one

**DOI:** 10.1107/S1600536810040079

**Published:** 2010-10-13

**Authors:** B. Palakshi Reddy, V. Vijayakumar, S. Sarveswari, Seik Weng Ng, Edward R. T. Tiekink

**Affiliations:** a Organic Chemistry Division, School of Advanced Sciences, VIT University, Vellore 632 014, India; b Department of Chemistry, University of Malaya, 50603 Kuala Lumpur, Malaysia

## Abstract

Each of the cyclohexenone rings in the title compound, C_23_H_27_NO_6_, adopts a half-chair (envelope) conformation with the C atom carrying the methyl groups lying out of the plane defined by the five remaining C atoms; the O atoms lie to the same side of the mol­ecule as the respective >C(CH_3_)_2_ atoms. The hy­droxy and carbonyl O atoms face each other and are orientated to allow for the formation of two intra­molecular O—H⋯O hydrogen bonds. In the crystal, the presence of C—H⋯O contacts leads to the formation of supra­molecular chains along the *b* axis. These aggregate into layers that stack along *c*.

## Related literature

For the biological activity and uses of xanthenes, see: Jonathan *et al.* (1988 ▶); Pohlers & Scaiano (1997 ▶); Hilderbrand & Weissleder (2007 ▶). For background to xanthenedione derivatives, see: Hatakeyama *et al.* (1988 ▶); Shchekotikhin & Nikolaeva (2006 ▶).
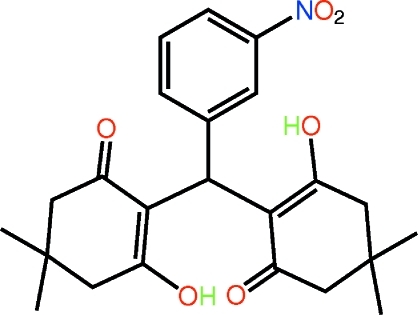


## Experimental

### 

#### Crystal data

C_23_H_27_NO_6_
*M* *_r_* = 413.46
Monoclinic, 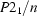

*a* = 14.2326 (10) Å
*b* = 8.6505 (6) Å
*c* = 16.8410 (12) Å
β = 97.796 (1)°
*V* = 2054.3 (3) Å^3^
*Z* = 4
Mo *K*α radiation
μ = 0.10 mm^−1^
*T* = 100 K
0.30 × 0.30 × 0.20 mm

#### Data collection

Bruker SMART APEX CCD diffractometer
Absorption correction: multi-scan (*SADABS*; Sheldrick, 1996 ▶) *T*_min_ = 0.792, *T*_max_ = 0.862
18883 measured reflections
4717 independent reflections
3928 reflections with *I* > 2σ(*I*)
*R*_int_ = 0.036

#### Refinement

*R*[*F* ^2^ > 2σ(*F* ^2^)] = 0.039
*wR*(*F* ^2^) = 0.112
*S* = 1.03
4717 reflections
279 parameters
2 restraints
H atoms treated by a mixture of independent and constrained refinement
Δρ_max_ = 0.37 e Å^−3^
Δρ_min_ = −0.23 e Å^−3^

### 

Data collection: *APEX2* (Bruker, 2008 ▶); cell refinement: *SAINT* (Bruker, 2008 ▶); data reduction: *SAINT*; program(s) used to solve structure: *SHELXS97* (Sheldrick, 2008 ▶); program(s) used to refine structure: *SHELXL97* (Sheldrick, 2008 ▶); molecular graphics: *ORTEP-3* (Farrugia, 1997 ▶) and *DIAMOND* (Brandenburg, 2006 ▶); software used to prepare material for publication: *publCIF* (Westrip, 2010 ▶).

## Supplementary Material

Crystal structure: contains datablocks global, I. DOI: 10.1107/S1600536810040079/hb5662sup1.cif

Structure factors: contains datablocks I. DOI: 10.1107/S1600536810040079/hb5662Isup2.hkl

Additional supplementary materials:  crystallographic information; 3D view; checkCIF report

## Figures and Tables

**Table 1:** Hydrogen-bond geometry (Å, °)

*D*—H⋯*A*	*D*—H	H⋯*A*	*D*⋯*A*	*D*—H⋯*A*
O1—H1*o*⋯O4	0.85 (1)	1.80 (1)	2.6392 (13)	167 (2)
O3—H3*o*⋯O2	0.86 (1)	1.75 (1)	2.5985 (13)	170 (2)
C5—H5a⋯O4^i^	0.99	2.34	3.2781 (16)	158
C9—H9⋯O6^ii^	1.00	2.56	3.3431 (16)	135
C21—H21⋯O2^iii^	0.95	2.44	3.3121 (16)	152

Symmetry codes: (i) 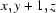; (ii) 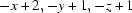; (iii) 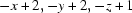.

**Fig. 1.**
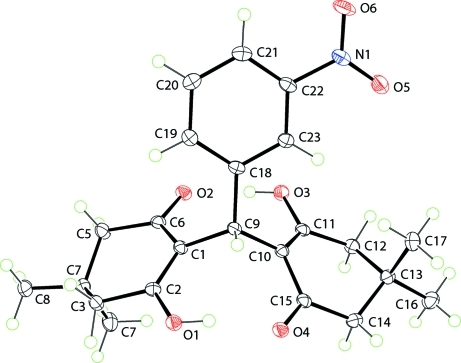
Molecular structure of (I) showing displacement ellipsoids at the 50% probability level.

**Fig. 2.**
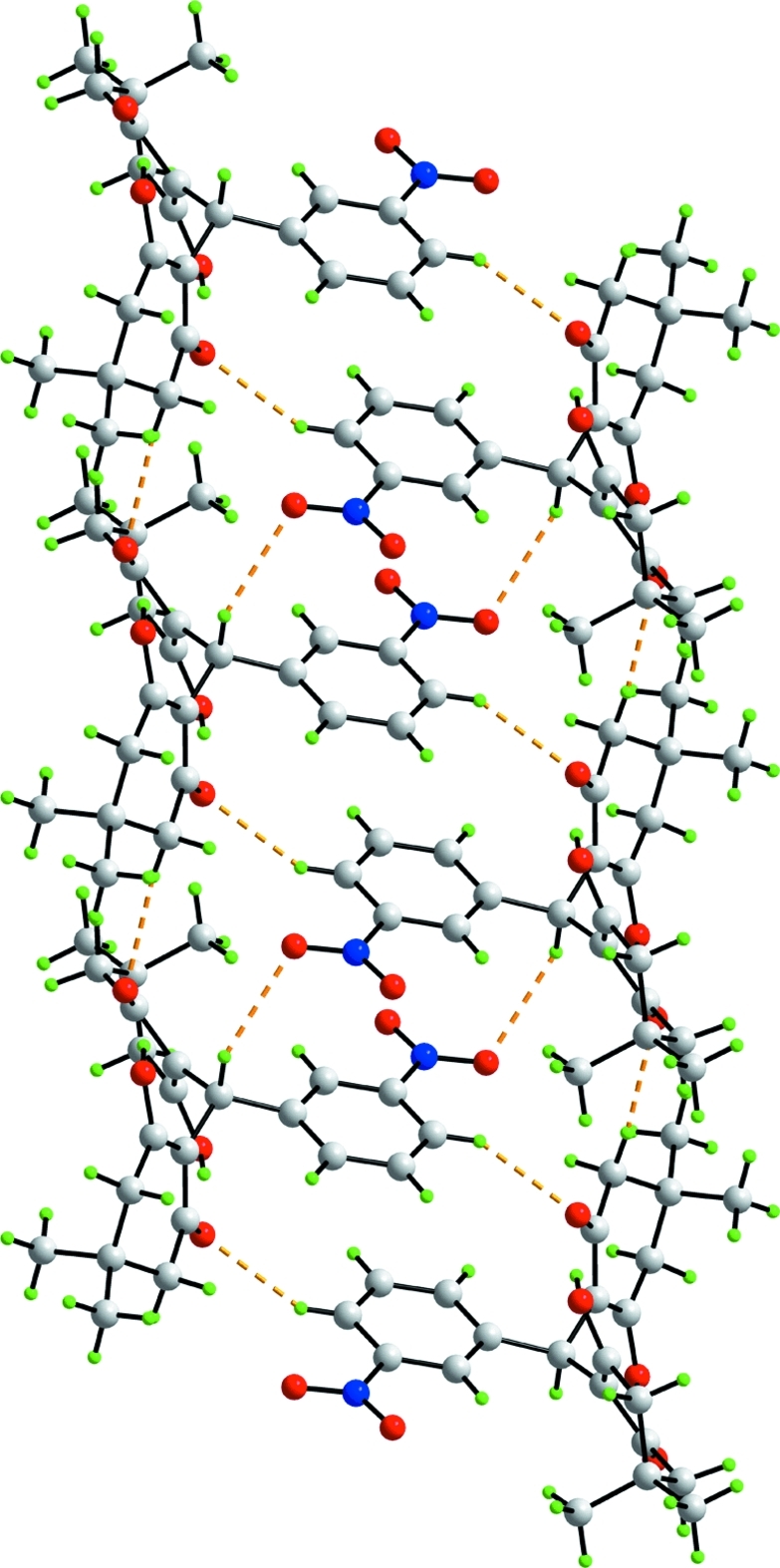
Supramolecular chain aligned along the b axis sustained by C—H···O contacts (shown as orange dashed lines).

**Fig. 3.**
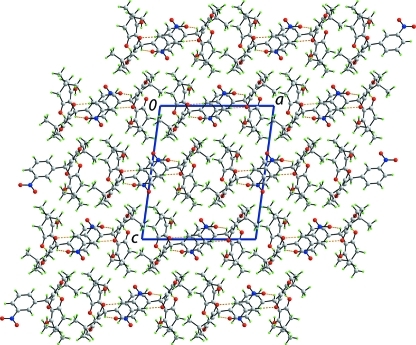
Unit-cell contents for (I) viewed in projection along the b axis showing the stacking of layers along the c axis. The C—H···O contacts are shown as orange dashed lines.

## supplementary crystallographic information

### Comment

Xanthenes are known for to possess various biological properties including anti-bacterial, anti-viral and anti-inflammatory activities (Jonathan *et al.*, 1988). In particular, xanthenedione derivatives constitute a structural unit in several natural products (Hatakeyama *et al.*, 1988), and they are valuable synthons because of the inherent reactivity of the in-built pyran ring (Shchekotikhin & Nikolaeva, 2006). Xanthene derivatives are also very useful and important organic compounds widely used as dyes (Hilderbrand & Weissleder, 2007), in laser technologies, and as fluorescent materials for visualization of biomolecules (Pohlers & Scaiano, 1997).

The molecular structure of the title compound, Fig. 1, features two cyclohexene rings, each with a half-chair conformation as, in each case, the C4 and C13 atoms, *i.e*. carrying two methyl groups, lie above the respective least-squares plane through the remaining five carbon atoms. For each ring, the O atoms lie to the same side of the molecule as the C4 or C13 atoms. The hydroxyl- and carbonyl-O atoms of one cyclohexene ring face the carbonyl- and hydroxyl-O atoms of the other to allow for the formation of intramolecular O—H···O hydrogen bonds, Table 1. The nitro group is co-planar with the benzene ring to which it is attached as seen in the O5—N1—C22—C21 torsion angle of -176.02 (11) °. The nitro-substituted benzene ring occupies a position almost side-on to both cyclohexene rings.

The most prominent intermolecular interactions in the crystal packing are of the type C—H···O, Table 1. These serve to link molecules into a supramolecular chain along the *b* axis, Fig. 2. The chains pack into layers in the *ab* plane which stack along the *c* axis, Fig. 3.

### Experimental

A mixture of 3-nitrobenaldehyde (0.377 g, 0.0025 mol), dimedone (0.7 g, 0.005 mol) was refluxed in acetonitrile (20 ml) for 3 h. The progress of the reaction was monitored by TLC. After completion of the reaction, the solution was left for 2 days to precipitate the solid product. The product was recrystallized by slow evaporation of its acetonitrile solution which yielded colourless blocks of (I). Yield: 72%. *M*.pt. 443–445 K.

### Refinement

Carbon-bound H-atoms were placed in calculated positions (C—H 0.95 to 1.00 Å) and were included in the refinement in the riding model approximation, with *U*_iso_(H) set to 1.2 to 1.5*U*_equiv_(C). The O-bound H atoms were refined with the distance restraint O—H = 0.84±0.1 Å, and with *U*_iso_(H) = 1.5*U*_equiv_(O).

### Crystal data

**Table:**

C_23_H_27_NO_6_	*F*(000) = 880
*M**_r_* = 413.46	*D*_x_ = 1.337 Mg m^−^^3^
Monoclinic, *P*2_1_/*n*	Mo *K*α radiation, λ = 0.71073 Å
Hall symbol: -P 2yn	Cell parameters from 5854 reflections
*a* = 14.2326 (10) Å	θ = 4.5–28.2°
*b* = 8.6505 (6) Å	µ = 0.10 mm^−^^1^
*c* = 16.8410 (12) Å	*T* = 100 K
β = 97.796 (1)°	Block, colourless
*V* = 2054.3 (3) Å^3^	0.30 × 0.30 × 0.20 mm
*Z* = 4	

### Data collection

**Table:**

Bruker SMART APEX CCD diffractometer	4717 independent reflections
Radiation source: fine-focus sealed tube	3928 reflections with *I* > 2σ(*I*)
graphite	*R*_int_ = 0.036
ω scans	θ_max_ = 27.5°, θ_min_ = 2.0°
Absorption correction: multi-scan (*SADABS*; Sheldrick, 1996)	*h* = −16→18
*T*_min_ = 0.792, *T*_max_ = 0.862	*k* = −11→11
18883 measured reflections	*l* = −21→21

### Refinement

**Table:**

Refinement on *F*^2^	Primary atom site location: structure-invariant direct methods
Least-squares matrix: full	Secondary atom site location: difference Fourier map
*R*[*F*^2^ > 2σ(*F*^2^)] = 0.039	Hydrogen site location: inferred from neighbouring sites
*wR*(*F*^2^) = 0.112	H atoms treated by a mixture of independent and constrained refinement
*S* = 1.03	*w* = 1/[σ^2^(*F*_o_^2^) + (0.0605*P*)^2^ + 0.67*P*] where *P* = (*F*_o_^2^ + 2*F*_c_^2^)/3
4717 reflections	(Δ/σ)_max_ = 0.001
279 parameters	Δρ_max_ = 0.37 e Å^−^^3^
2 restraints	Δρ_min_ = −0.23 e Å^−^^3^

### Special details

**Table:**

Geometry. All e.s.d.'s (except the e.s.d. in the dihedral angle between two l.s. planes) are estimated using the full covariance matrix. The cell e.s.d.'s are taken into account individually in the estimation of e.s.d.'s in distances, angles and torsion angles; correlations between e.s.d.'s in cell parameters are only used when they are defined by crystal symmetry. An approximate (isotropic) treatment of cell e.s.d.'s is used for estimating e.s.d.'s involving l.s. planes.
Refinement. Refinement of *F*^2^ against ALL reflections. The weighted *R*-factor *wR* and goodness of fit *S* are based on *F*^2^, conventional *R*-factors *R* are based on *F*, with *F* set to zero for negative *F*^2^. The threshold expression of *F*^2^ > σ(*F*^2^) is used only for calculating *R*-factors(gt) *etc*. and is not relevant to the choice of reflections for refinement. *R*-factors based on *F*^2^ are statistically about twice as large as those based on *F*, and *R*- factors based on ALL data will be even larger.

### Fractional atomic coordinates and isotropic or equivalent isotropic displacement parameters (Å^2^)

**Table:**

	*x*	*y*	*z*	*U*_iso_*/*U*_eq_	
O1	0.65593 (7)	0.65589 (10)	0.67564 (5)	0.0159 (2)	
H1o	0.6569 (15)	0.594 (2)	0.6365 (9)	0.048 (6)*	
O2	0.73862 (7)	1.02234 (10)	0.49100 (5)	0.0170 (2)	
O3	0.73479 (7)	0.82683 (11)	0.37346 (5)	0.0170 (2)	
H3o	0.7407 (13)	0.8846 (19)	0.4154 (8)	0.036 (5)*	
O4	0.63116 (6)	0.46796 (10)	0.55110 (5)	0.0163 (2)	
O5	0.99653 (7)	0.53999 (12)	0.35663 (6)	0.0237 (2)	
O6	1.13360 (7)	0.63202 (12)	0.40630 (6)	0.0259 (2)	
N1	1.04746 (8)	0.61823 (13)	0.40582 (7)	0.0176 (2)	
C1	0.71426 (9)	0.83167 (14)	0.58531 (7)	0.0129 (2)	
C2	0.67059 (9)	0.79995 (14)	0.65085 (7)	0.0134 (2)	
C3	0.63844 (9)	0.92120 (14)	0.70461 (7)	0.0153 (3)	
H3A	0.6898	0.9405	0.7493	0.018*	
H3B	0.5829	0.8817	0.7279	0.018*	
C4	0.61160 (9)	1.07463 (14)	0.66175 (7)	0.0149 (3)	
C5	0.69030 (10)	1.11682 (15)	0.61177 (8)	0.0184 (3)	
H5A	0.6701	1.2088	0.5788	0.022*	
H5B	0.7480	1.1453	0.6486	0.022*	
C6	0.71497 (9)	0.98965 (15)	0.55741 (8)	0.0145 (3)	
C7	0.51615 (10)	1.05773 (17)	0.60799 (8)	0.0217 (3)	
H7A	0.4669	1.0306	0.6409	0.032*	
H7B	0.5209	0.9761	0.5684	0.032*	
H7C	0.4997	1.1557	0.5803	0.032*	
C8	0.60339 (10)	1.20199 (15)	0.72332 (8)	0.0187 (3)	
H8A	0.5531	1.1754	0.7554	0.028*	
H8B	0.5880	1.3002	0.6955	0.028*	
H8C	0.6638	1.2121	0.7585	0.028*	
C9	0.76179 (8)	0.70610 (14)	0.54208 (7)	0.0124 (2)	
H9	0.7692	0.6185	0.5812	0.015*	
C10	0.70261 (9)	0.63781 (14)	0.46833 (7)	0.0129 (2)	
C11	0.69851 (9)	0.69205 (14)	0.39210 (8)	0.0139 (3)	
C12	0.65447 (9)	0.60340 (15)	0.31987 (8)	0.0162 (3)	
H12A	0.6947	0.6153	0.2767	0.019*	
H12B	0.5916	0.6487	0.3006	0.019*	
C13	0.64204 (9)	0.43071 (15)	0.33584 (8)	0.0162 (3)	
C14	0.59406 (10)	0.41744 (16)	0.41182 (8)	0.0189 (3)	
H14A	0.5281	0.4556	0.3999	0.023*	
H14B	0.5913	0.3070	0.4268	0.023*	
C15	0.64394 (9)	0.50637 (14)	0.48217 (7)	0.0140 (2)	
C16	0.57961 (10)	0.35702 (17)	0.26472 (8)	0.0228 (3)	
H16A	0.5718	0.2467	0.2752	0.034*	
H16B	0.6097	0.3698	0.2161	0.034*	
H16C	0.5174	0.4074	0.2573	0.034*	
C17	0.73790 (10)	0.34775 (15)	0.34742 (8)	0.0201 (3)	
H17A	0.7282	0.2378	0.3577	0.030*	
H17B	0.7786	0.3932	0.3931	0.030*	
H17C	0.7683	0.3592	0.2989	0.030*	
C18	0.86411 (8)	0.74648 (14)	0.53038 (7)	0.0130 (2)	
C19	0.91956 (9)	0.84390 (15)	0.58406 (8)	0.0156 (3)	
H19	0.8912	0.8949	0.6248	0.019*	
C20	1.01514 (9)	0.86788 (15)	0.57927 (8)	0.0175 (3)	
H20	1.0509	0.9350	0.6165	0.021*	
C21	1.05918 (9)	0.79508 (15)	0.52075 (8)	0.0171 (3)	
H21	1.1245	0.8105	0.5170	0.021*	
C22	1.00352 (9)	0.69910 (15)	0.46819 (7)	0.0150 (3)	
C23	0.90774 (9)	0.67322 (14)	0.47154 (7)	0.0144 (3)	
H23	0.8724	0.6062	0.4340	0.017*	

### Atomic displacement parameters (Å^2^)

**Table:**

	*U*^11^	*U*^22^	*U*^33^	*U*^12^	*U*^13^	*U*^23^
O1	0.0196 (5)	0.0135 (4)	0.0154 (4)	−0.0009 (4)	0.0058 (4)	0.0015 (3)
O2	0.0193 (5)	0.0155 (5)	0.0174 (4)	−0.0004 (4)	0.0067 (4)	0.0025 (3)
O3	0.0208 (5)	0.0146 (4)	0.0161 (5)	−0.0015 (4)	0.0048 (4)	0.0019 (4)
O4	0.0186 (5)	0.0138 (4)	0.0176 (4)	−0.0009 (3)	0.0067 (4)	0.0010 (3)
O5	0.0225 (5)	0.0281 (5)	0.0216 (5)	0.0016 (4)	0.0070 (4)	−0.0041 (4)
O6	0.0152 (5)	0.0308 (6)	0.0342 (6)	0.0022 (4)	0.0126 (4)	0.0012 (5)
N1	0.0169 (6)	0.0171 (5)	0.0204 (6)	0.0040 (4)	0.0078 (4)	0.0052 (4)
C1	0.0115 (6)	0.0124 (6)	0.0151 (6)	0.0015 (4)	0.0027 (5)	0.0000 (4)
C2	0.0113 (6)	0.0141 (6)	0.0146 (6)	0.0008 (5)	0.0012 (4)	0.0017 (5)
C3	0.0167 (6)	0.0159 (6)	0.0140 (6)	0.0006 (5)	0.0048 (5)	−0.0002 (5)
C4	0.0151 (6)	0.0155 (6)	0.0142 (6)	0.0017 (5)	0.0029 (5)	−0.0015 (5)
C5	0.0225 (7)	0.0127 (6)	0.0216 (6)	0.0015 (5)	0.0091 (5)	0.0001 (5)
C6	0.0113 (6)	0.0149 (6)	0.0176 (6)	0.0000 (5)	0.0035 (5)	0.0001 (5)
C7	0.0183 (7)	0.0257 (7)	0.0201 (7)	0.0054 (5)	−0.0003 (5)	−0.0049 (5)
C8	0.0190 (6)	0.0175 (6)	0.0198 (6)	0.0019 (5)	0.0042 (5)	−0.0034 (5)
C9	0.0123 (6)	0.0120 (6)	0.0134 (6)	0.0007 (4)	0.0036 (4)	0.0010 (4)
C10	0.0106 (5)	0.0126 (6)	0.0158 (6)	0.0013 (5)	0.0029 (5)	−0.0004 (5)
C11	0.0110 (6)	0.0130 (6)	0.0183 (6)	0.0019 (4)	0.0045 (5)	0.0010 (5)
C12	0.0159 (6)	0.0179 (6)	0.0149 (6)	0.0005 (5)	0.0021 (5)	0.0010 (5)
C13	0.0161 (6)	0.0173 (6)	0.0153 (6)	−0.0027 (5)	0.0028 (5)	−0.0012 (5)
C14	0.0181 (6)	0.0199 (7)	0.0194 (6)	−0.0062 (5)	0.0051 (5)	−0.0017 (5)
C15	0.0120 (6)	0.0133 (6)	0.0173 (6)	0.0029 (5)	0.0042 (5)	0.0002 (5)
C16	0.0231 (7)	0.0258 (7)	0.0191 (7)	−0.0059 (6)	0.0012 (5)	−0.0037 (6)
C17	0.0226 (7)	0.0164 (6)	0.0214 (7)	0.0025 (5)	0.0034 (5)	−0.0026 (5)
C18	0.0118 (6)	0.0134 (6)	0.0145 (6)	0.0019 (5)	0.0036 (5)	0.0047 (4)
C19	0.0167 (6)	0.0157 (6)	0.0145 (6)	0.0018 (5)	0.0028 (5)	0.0015 (5)
C20	0.0162 (6)	0.0170 (6)	0.0185 (6)	−0.0020 (5)	−0.0005 (5)	0.0020 (5)
C21	0.0131 (6)	0.0175 (6)	0.0209 (6)	0.0007 (5)	0.0030 (5)	0.0059 (5)
C22	0.0142 (6)	0.0145 (6)	0.0173 (6)	0.0039 (5)	0.0058 (5)	0.0049 (5)
C23	0.0145 (6)	0.0138 (6)	0.0152 (6)	0.0005 (5)	0.0030 (5)	0.0023 (5)

### Geometric parameters (Å, °)

**Table:**

O1—C2	1.3395 (15)	C9—H9	1.0000
O1—H1o	0.851 (9)	C10—C11	1.3607 (17)
O2—C6	1.2436 (15)	C10—C15	1.4481 (17)
O3—C11	1.3299 (15)	C11—C12	1.5014 (18)
O3—H3o	0.860 (9)	C12—C13	1.5323 (18)
O4—C15	1.2443 (15)	C12—H12A	0.9900
O5—N1	1.2275 (15)	C12—H12B	0.9900
O6—N1	1.2308 (14)	C13—C17	1.5304 (18)
N1—C22	1.4704 (16)	C13—C16	1.5303 (18)
C1—C2	1.3662 (17)	C13—C14	1.5343 (18)
C1—C6	1.4456 (17)	C14—C15	1.5072 (18)
C1—C9	1.5169 (16)	C14—H14A	0.9900
C2—C3	1.4971 (17)	C14—H14B	0.9900
C3—C4	1.5341 (17)	C16—H16A	0.9800
C3—H3A	0.9900	C16—H16B	0.9800
C3—H3B	0.9900	C16—H16C	0.9800
C4—C8	1.5282 (17)	C17—H17A	0.9800
C4—C5	1.5337 (18)	C17—H17B	0.9800
C4—C7	1.5340 (18)	C17—H17C	0.9800
C5—C6	1.5029 (17)	C18—C23	1.3913 (17)
C5—H5A	0.9900	C18—C19	1.3988 (18)
C5—H5B	0.9900	C19—C20	1.3893 (18)
C7—H7A	0.9800	C19—H19	0.9500
C7—H7B	0.9800	C20—C21	1.3886 (19)
C7—H7C	0.9800	C20—H20	0.9500
C8—H8A	0.9800	C21—C22	1.3821 (19)
C8—H8B	0.9800	C21—H21	0.9500
C8—H8C	0.9800	C22—C23	1.3902 (18)
C9—C10	1.5223 (17)	C23—H23	0.9500
C9—C18	1.5360 (16)		
			
C2—O1—H1o	109.0 (14)	O3—C11—C12	112.91 (11)
C11—O3—H3o	108.3 (13)	C10—C11—C12	123.24 (11)
O6—N1—O5	123.66 (11)	C11—C12—C13	113.65 (10)
O6—N1—C22	117.97 (11)	C11—C12—H12A	108.8
O5—N1—C22	118.36 (11)	C13—C12—H12A	108.8
C2—C1—C6	118.45 (11)	C11—C12—H12B	108.8
C2—C1—C9	121.69 (11)	C13—C12—H12B	108.8
C6—C1—C9	119.85 (11)	H12A—C12—H12B	107.7
O1—C2—C1	123.08 (11)	C17—C13—C16	108.50 (11)
O1—C2—C3	112.96 (10)	C17—C13—C12	110.94 (11)
C1—C2—C3	123.90 (11)	C16—C13—C12	109.71 (11)
C2—C3—C4	113.50 (10)	C17—C13—C14	110.40 (11)
C2—C3—H3A	108.9	C16—C13—C14	110.16 (11)
C4—C3—H3A	108.9	C12—C13—C14	107.12 (11)
C2—C3—H3B	108.9	C15—C14—C13	113.70 (11)
C4—C3—H3B	108.9	C15—C14—H14A	108.8
H3A—C3—H3B	107.7	C13—C14—H14A	108.8
C8—C4—C5	109.24 (11)	C15—C14—H14B	108.8
C8—C4—C7	109.03 (11)	C13—C14—H14B	108.8
C5—C4—C7	110.68 (11)	H14A—C14—H14B	107.7
C8—C4—C3	109.97 (10)	O4—C15—C10	121.35 (12)
C5—C4—C3	108.08 (10)	O4—C15—C14	118.96 (11)
C7—C4—C3	109.83 (11)	C10—C15—C14	119.65 (11)
C6—C5—C4	114.11 (11)	C13—C16—H16A	109.5
C6—C5—H5A	108.7	C13—C16—H16B	109.5
C4—C5—H5A	108.7	H16A—C16—H16B	109.5
C6—C5—H5B	108.7	C13—C16—H16C	109.5
C4—C5—H5B	108.7	H16A—C16—H16C	109.5
H5A—C5—H5B	107.6	H16B—C16—H16C	109.5
O2—C6—C1	121.46 (12)	C13—C17—H17A	109.5
O2—C6—C5	119.71 (11)	C13—C17—H17B	109.5
C1—C6—C5	118.79 (11)	H17A—C17—H17B	109.5
C4—C7—H7A	109.5	C13—C17—H17C	109.5
C4—C7—H7B	109.5	H17A—C17—H17C	109.5
H7A—C7—H7B	109.5	H17B—C17—H17C	109.5
C4—C7—H7C	109.5	C23—C18—C19	117.87 (11)
H7A—C7—H7C	109.5	C23—C18—C9	120.68 (11)
H7B—C7—H7C	109.5	C19—C18—C9	121.04 (11)
C4—C8—H8A	109.5	C20—C19—C18	121.57 (12)
C4—C8—H8B	109.5	C20—C19—H19	119.2
H8A—C8—H8B	109.5	C18—C19—H19	119.2
C4—C8—H8C	109.5	C19—C20—C21	120.88 (12)
H8A—C8—H8C	109.5	C19—C20—H20	119.6
H8B—C8—H8C	109.5	C21—C20—H20	119.6
C1—C9—C10	115.85 (10)	C22—C21—C20	116.90 (12)
C1—C9—C18	113.02 (10)	C22—C21—H21	121.6
C10—C9—C18	114.39 (10)	C20—C21—H21	121.6
C1—C9—H9	103.9	C21—C22—C23	123.42 (12)
C10—C9—H9	103.9	C21—C22—N1	118.75 (11)
C18—C9—H9	103.9	C23—C22—N1	117.83 (11)
C11—C10—C15	118.08 (11)	C22—C23—C18	119.37 (12)
C11—C10—C9	125.74 (11)	C22—C23—H23	120.3
C15—C10—C9	116.15 (10)	C18—C23—H23	120.3
O3—C11—C10	123.83 (12)		
			
C6—C1—C2—O1	171.69 (11)	C10—C11—C12—C13	−17.54 (17)
C9—C1—C2—O1	−8.61 (19)	C11—C12—C13—C17	−71.32 (14)
C6—C1—C2—C3	−11.38 (18)	C11—C12—C13—C16	168.81 (11)
C9—C1—C2—C3	168.32 (11)	C11—C12—C13—C14	49.24 (14)
O1—C2—C3—C4	−153.63 (11)	C17—C13—C14—C15	68.28 (14)
C1—C2—C3—C4	29.16 (17)	C16—C13—C14—C15	−171.90 (11)
C2—C3—C4—C8	−166.16 (11)	C12—C13—C14—C15	−52.62 (14)
C2—C3—C4—C5	−46.99 (14)	C11—C10—C15—O4	−167.09 (11)
C2—C3—C4—C7	73.84 (14)	C9—C10—C15—O4	11.17 (17)
C8—C4—C5—C6	171.38 (11)	C11—C10—C15—C14	10.51 (18)
C7—C4—C5—C6	−68.56 (14)	C9—C10—C15—C14	−171.23 (11)
C3—C4—C5—C6	51.74 (14)	C13—C14—C15—O4	−157.72 (12)
C2—C1—C6—O2	−167.47 (12)	C13—C14—C15—C10	24.63 (17)
C9—C1—C6—O2	12.83 (18)	C1—C9—C18—C23	−159.11 (11)
C2—C1—C6—C5	15.06 (18)	C10—C9—C18—C23	−23.66 (16)
C9—C1—C6—C5	−164.65 (11)	C1—C9—C18—C19	28.36 (16)
C4—C5—C6—O2	145.36 (12)	C10—C9—C18—C19	163.81 (11)
C4—C5—C6—C1	−37.13 (16)	C23—C18—C19—C20	0.13 (18)
C2—C1—C9—C10	96.76 (14)	C9—C18—C19—C20	172.86 (11)
C6—C1—C9—C10	−83.54 (14)	C18—C19—C20—C21	−0.2 (2)
C2—C1—C9—C18	−128.46 (12)	C19—C20—C21—C22	0.12 (19)
C6—C1—C9—C18	51.23 (15)	C20—C21—C22—C23	−0.03 (19)
C1—C9—C10—C11	88.93 (15)	C20—C21—C22—N1	−179.31 (11)
C18—C9—C10—C11	−45.23 (16)	O6—N1—C22—C21	4.50 (17)
C1—C9—C10—C15	−89.18 (13)	O5—N1—C22—C21	−176.02 (11)
C18—C9—C10—C15	136.66 (11)	O6—N1—C22—C23	−174.82 (11)
C15—C10—C11—O3	167.35 (11)	O5—N1—C22—C23	4.65 (17)
C9—C10—C11—O3	−10.7 (2)	C21—C22—C23—C18	−0.02 (19)
C15—C10—C11—C12	−14.28 (18)	N1—C22—C23—C18	179.27 (11)
C9—C10—C11—C12	167.64 (11)	C19—C18—C23—C22	−0.03 (18)
O3—C11—C12—C13	160.99 (11)	C9—C18—C23—C22	−172.79 (11)

### Hydrogen-bond geometry (Å, °)

**Table:**

*D*—H···*A*	*D*—H	H···*A*	*D*···*A*	*D*—H···*A*
O1—H1o···O4	0.85 (1)	1.80 (1)	2.6392 (13)	167 (2)
O3—H3o···O2	0.86 (1)	1.75 (1)	2.5985 (13)	170 (2)
C5—H5a···O4^i^	0.99	2.34	3.2781 (16)	158
C9—H9···O6^ii^	1.00	2.56	3.3431 (16)	135
C21—H21···O2^iii^	0.95	2.44	3.3121 (16)	152

Symmetry codes: (i) *x*, *y*+1, *z*; (ii) −*x*+2, −*y*+1, −*z*+1; (iii) −*x*+2, −*y*+2, −*z*+1.

## References

[bb1] Brandenburg, K. (2006). *DIAMOND* Crystal Impact GbR, Bonn, Germany.

[bb2] Bruker (2008). *APEX2* and *SAINT* Bruker AXS Inc., Madison, Wisconsin, USA.

[bb3] Farrugia, L. J. (1997). *J. Appl. Cryst.* **30**, 565.

[bb4] Hatakeyama, S., Ochi, N., Numata, H. & Takano, S. (1988). *Chem. Commun.* pp. 1202–1204.

[bb5] Hilderbrand, S. A. & Weissleder, R. (2007). *Tetrahedron Lett.* **48**, 4383–4385. 10.1016/j.tetlet.2007.04.088 PMC2761723 19834587

[bb6] Jonathan, R. D., Srinivas, K. R. & Glen, E. B. (1988). *Eur. J. Med. Chem.* **23**, 111–117.

[bb7] Pohlers, G. & Scaiano, J. C. (1997). *Chem. Mater.* **9**, 3222–3230.

[bb8] Shchekotikhin, Y. M. & Nikolaeva, T. G. (2006). *Chem. Heterocycl. Compd*, **42**, 28–33.

[bb9] Sheldrick, G. M. (1996). *SADABS* University of Göttingen, Germany.

[bb10] Sheldrick, G. M. (2008). *Acta Cryst.* A**64**, 112–122. 10.1107/S0108767307043930 18156677

[bb11] Westrip, S. P. (2010). *J. Appl. Cryst.* **43**, 920–925.

